# CDK 4/6 inhibitors for adjuvant therapy in early breast cancer—Do we have a clear winner?

**DOI:** 10.3332/ecancer.2022.ed124

**Published:** 2022-08-30

**Authors:** Amol Akhade, Simon Van Wambeke, Bishal Gyawali

**Affiliations:** 1 BYL Nair Hospital and TN Medical College, Mumbai, Maharashtra 400008, India; 2 Department of Oncology, ZNA, Antwerp B-2020, Belgium; 3 Departments of Oncology and Public Health Sciences, Queen’s University, Kingston K7L 3N6, Canada; 4 Queen’s Global Oncology Program, Queen’s University, Kingston K7L 3N6, Canada

**Keywords:** Abemaciclib, Palbociclib, Ribociclib, early stage breast cancer, Ki-67, disease-free survival, overall survival

## Abstract

CDK4/6 inhibitors have become the mainstay of treatment for patients with advanced hormone receptor positive and Human Epidermal Receptor -2 [ HER-2 ] negative breast cancer. Three CDK 4/6 inhibitor drugs are currently approved and available, including Palbociclib, Ribociclib and Abemaciclib. All three of these drugs have similar mechanism of action and other pharmacokinetic and pharmaco-dynamic properties and hold equivalent positions in cancer care guidelines. Surprisingly, however, in the adjuvant setting of early breast cancer, two trials of palbociclib have failed to show any benefit while abemaciclib has shown some early benefits in disease-free survival and has received approval for its use in adjuvant setting. In this article, we explore several reasons for this discrepancy in the results of CDK4/6 inhibitors in the adjuvant setting. We also question if we should already adopt adjuvant abemaciclib in our clinical practice given the uncertainty in data.

Cyclin–dependent kinase (CDK) 4/6 inhibitors in combination with endocrine therapy (ET) have become the mainstay of treatment for advanced hormone receptor (HR) positive breast cancer over the last few years. Palbociclib was the first CDK 4/6 inhibitor to receive the FDA approval as accelerated approval in 2015 and full approval in 2017 for HR positive and human epidermal receptor-2 (HER-2) negative metastatic breast cancer. Since then, two more CDK 4/6 inhibitors, ribociclib and abemaciclib have received full approval for the same indication. Although ribociclib is the only agent to show an overall survival (OS) benefit in the first line so far, there have been some concerns about lack of standard post-protocol treatment in the trial [1]. Furthermore, network meta-analysis has shown that the three CDK 4/6 inhibitors have no statistically significant differences between them [2]. Treatment guidelines such as the NCCN and ASCO now recommend combining any CDK 4/6 inhibitors with ET as standard first line treatment for patients with metastatic breast cancer with little to choose among the three [3].

However, interestingly, the results of the three CDK 4/6 inhibitors have differed when tested in the adjuvant setting. Thus, it is important to ask if these differences seen in the adjuvant setting are a play of statistics and trial design or if there actually is a meaningful difference among the CDK 4/6 inhibitors in the adjuvant setting.

In the PENELOPE-B trial, 1250 patients with early HR positive breast cancer at high risk of relapse who had no pathological complete response after neo-adjuvant chemotherapy were randomly assigned to one year of palbociclib vs placebo [4]. High-risk in this trial was defined as estrogen receptor grading score ≥ 3 or 2 with residual lymph-node positive. At a median follow up of 42.8 months, there was no difference between the treatment arms for the primary endpoint of invasive disease-free survival (iDFS) ( 3 year iDFS rates 81.2% v 77.3%, HR 0.93, p=0.525). The HR for patients whose tumors had high Ki-67 (>15%) was 1.020 while that for those with Ki-67 less than 15% was 0.87; this difference was not statistically significant. Therefore, Ki- 67 was not a predictor of a difference in iDFS outcomes in this trial.

In the PALLAS trial involving 5685 patients, patients with stage II or III HR positive breast cancer were randomized to adjuvant ET + 2 years of palbociclib versus placebo [5]. At the second interim analysis, iDFS was not improved and the trial was stopped for futility by the independent data monitoring committee (estimated 3y iDFS 89.3% vs 89.4%). Ki-67 status was not evaluated in this trial.

Abemaciclib was tested in the monarchE Trial that included 5637 patients with high-risk disease and comprised of two cohorts; cohort 1 enrolled patients with ≥4 positive axillary lymph nodes (ALNs), or 1-3 positive ALNs and either grade 3 disease or tumor ≥5 cm, and cohort 2 enrolled patients with 1-3 positive ALNs and high Ki-67 index (≥20%) [6]. Patients were randomized to adjuvant ET + 2 years of abemaciclib or placebo. After 19 months of median follow up, a statistically significant improvement in iDFS was observed (HR=0.71, p= 0.0009). Ki-67 was more than 20% in 44% of patients. Among 44% patients who had high Ki-67, the estimated 3y iDFS was 86.8 % vs 80.8 % (HR 0.663) whereas in the Ki-67 low cohort, it was 91.7 % vs 87.2 % (HR 0.70). Thus, although Ki-67 was not found to be predictive of response, it was found to be prognostic since the iDFS rates for low Ki-67 cohort was better than that for high Ki-67 cohort irrespective of treatment arm [7].

These results led to U.S. FDA approval of 2 years of adjuvant abemaciclib in patients with early HR positive breast cancer at a high risk for relapse, but only when Ki-67 ≥20%. The FDA limited its indication to Ki-67 ≥ 20% because in this sub-population of monarchE trial an iDFS benefit was seen without a potential detriment in overall survival (HR for OS was 0.767 ), however the HR for OS in the ITT population was 1.091, indicating potentially detrimental effect on OS among patients with low Ki-67. However, the European Medicines Agency (EMA) approved this indication without any Ki-67 based restrictions [8].

The results of the trial testing three years of adjuvant ribociclib in early breast cancer has not yet been reported.

The positive results of abemaciclib compared to the negative results of palbociclib in early breast cancer raises several questions. Why would two CDK 4/6 inhibitors with similar efficacy in metastatic setting have differential outcomes in adjuvant setting?

One proposed explanation is the selection of high-risk patients with positive lymph nodes in monarchE unlike PALLAS that included all stage II and III patients (Table 1). The percent of patients with stage III and grade 3 tumors were 48.9% and 29.3% respectively in PALLAS compared with 74.1% and 38.7%, respectively in monarchE. Therefore, some experts argue that PALLAS was negative simply because it did not recruit enough high-risk patients. However, an exploratory subgroup analysis from the PALLAS trial showed no advantage of Palbociclib in high-risk patients compared to lower-risk patients (HR of 0.89 vs HR of 0.93) [5]. In addition, Penelope-B included very high-risk patients and yet, there was no benefit for iDFS. Therefore, high-risk alone does not explain these differential outcomes.

Can drug exposure or duration of treatment explain these differences? Even though the rate of discontinuation due to drug related adverse events was similar (21.1% in PALLAS vs 17.2 % in monarchE), PALLAS had more drug stoppage intervals, due to neutropenia, as per trial protocol. In a post hoc analysis, no significant relationship was seen between longer palbociclib duration or exposure intensity and improved iDFS [5].

Finally, with the OS results of PALOMA-2 trial presented at ASCO 2022 Annual Meeting, it is also worthwhile asking if palbociclib truly is different among the CDK 4/6 inhibitors [9]. In PALOMA-2, palbociclib failed to improve OS in the advanced setting in the first-line despite improving PFS, a setting where ribociclib has proven OS benefits. However, more data are needed to determine if there is a genuine difference among the three CDK 4/6 inhibitors and whether this can explain the differences between palbociclib and abemaciclib results in the adjuvant setting.

Although most debates start with the premise of why palbociclib results were negative, it is equally important to think if it is the abemaciclib results that should be questioned. Could it be that PALLAS and PENELOPE are true negative but monarchE was a false positive? We are not suggesting that that is the case, but that it is also an equally plausible alternative. However, the question that matters the most in clinical practice is whether adjuvant ademaciclib should be the standard of care.

First, iDFS is not a valid surrogate for OS in early breast cancer, except in HER2 positive subgroup [10]. As of now, the immature OS data from monarchE show no benefit for the experimental arm with the HR favouring the control arm instead. Without an OS benefit, using CDK 4/6 inhibitors as adjuvant therapy may simply jeopardize the patients’ chances of benefitting from the same medication if and when they relapse. Many of these patients may not relapse. Thus, we would be subjecting patients to an expensive treatment and increase the therapeutic burden without knowing it will make any positive difference in their overall survival, adding toxicity, and jeopardizing their shot at an effective treatment during relapse. In theory﻿, a better risk stratification tool using genomic assays may tilt the balance towards identification and treatment of high-risk patients. However, no such tools are currently available either in metastatic or the adjuvant setting.

Furthermore, we need to ensure that patients in the control arm of monarchE have received CDK 4/6 inhibitors at the time of relapse. If they have received a CDK 4/6 inhibitor at relapse and achieve similar OS, that means irrespective of iDFS results, patients could very well reserve the drug to be used at the time of relapse. If patients in the control arm haven’t received CDK4/6 inhibitors at relapse, even OS gains do not prove early receipt of CDK 4/6 inhibitors is better than receiving them at the time of relapse. The possibility of a worse OS also exists, if early CDK 4/6 exposure leads to the development of an aggressive, treatment resistant clone. Given the HR for OS favoring the control arm in the ITT population, the FDA restricted the use of abemaciclib, based on an exploratory analysis, to Ki-67 high patients.

The FDA approval of abemaciclib based upon Ki-67 more than 20% happens to be first of its kind. Ki-67 is not a genomic marker or a target of abemaciclib. Ki-67 is a marker of cellular proliferation. It is a prognostic marker for early-stage breast cancer and not a predictive marker for treatment efficacy [11]. There are no studies that have shown that Ki-67 alone can be used as a predictor of benefit for CDK inhibitors or any other drug in early or advanced stage breast cancer. Ki-67 is used to assist determination of grades in other malignancies such as neuroendocrine tumors or colorectal cancers but has never been used as a sole criterion for therapeutic decisions. Indeed, tumors at a high risk of relapse can have low Ki67 at the time of presentation while otherwise low-risk tumors may have a high Ki-67.

Second, the follow-up time is too short for an adjuvant breast cancer trial with assessment of the primary outcome at only 19 months of median follow up, with only 25% of patients having completed their 2-year CDK 4/6 inhibitor treatment. An additional follow-up analysis, conducted at regulatory request performed at 27 months (with 90% of patients having either completed or prematurely discontinued their the 2-year treatment) confirmed the iDFS benefit but with no OS gains. This is still a short follow-up. In PenelopeB, iDFS benefit was seen at 2-3 years, similar to monarchE, only for the iDFS curves to converge at the final analysis at 42.8 months. We do not understand the haste in approving an agent in adjuvant setting and including it in guidelines based on premature data and dubious companion diagnostic when other agents of the same class have produced negative results.

Third, the protocol of monarchE did not specify the imaging modality to be used for staging of visceral disease. Some patients might thus have had subclinical metastatic disease, where we already know CDK 4-6 inhibitors are effective.

Fourth, the addition of abemaciclib to ET results in substantial side effects. With abemaciclib, patients experienced a higher incidence of Grade≥3 AEs (49.7% vs 16.3%). In adjuvant setting, compared with placebo, one can only produce detrimental, or at best stable, effects on QoL while on treatment. The frequency of PRO assessments in monarchE was insufficient to capture patient-reported side effects and QoL within the first 3 months.

Finally, the cost of 2 years of abemaciclib is estimated at >300,000 USD. That’s a substantial financial toxicity on health care system for a drug with uncertain benefit but known toxicities.

Even if abemaciclib is declared a winner in early breast cancer receiving the nod from the FDA and EMA based on very early positive iDFS results, we urge caution before accepting this as a routine standard of care by the guidelines and the breast cancer community. Considering the added therapeutic burden for 2 years, the jeopardizing of an important treatment option at relapse, the adverse effects, the financial toxicity to the patients and healthcare system, and negative results from other two trials of a similar class of drug, we ask the breast cancer community if it’s really worth rushing to prescribe this drug in adjuvant setting without waiting for mature survival results?

## Conclusion

When the results from two trials are conflicting, one should question not only if the negative trial is a false negative, but also whether the positive trial is a false positive. In the case of adjuvant trials of CDK 4/6 inhibitors in early breast cancer, even the “positive trial” of abemaciclib is marginally positive for an unvalidated surrogate endpoint at a very early look of the data. Given the potential for overtreating several patients for a dubious or even non-existent benefit in a small percentage of patients, we believe it is premature to recommend adjuvant abemaciclib to patients with early breast cancer without waiting for mature data.

## Conflicts of interest

Dr Van Wambeke has received speaker fees from Janssen and IPSEN. Dr Gyawali reports receiving consulting fees from Vivio Health, outside of the submitted work. No other conflicts of interest to disclose.

## Figures and Tables

**Table 1.** Comparison of the three adjuvant trials of CDK 4/6 inhibitors in early breast cancer

	PENELOPE-B trial	PALLAS trial	MonarchE Trial
Number of patients enrolled	1250	5685	5637
Inclusion criteria	Hormone receptor positive, HER2 negative early breast cancer without pathological complete response post neo-adjuvant therapy and at a high risk of relapse. High risk of relapse defined as clinical pathological staging-estrogen receptor grading score ≥ 3 or 2 and ypN+	Hormone receptor positive, HER2 negative stage II or III cancer	Hormone receptor positive, HER2 negative disease at high risk of relapse. High risk defined as: More than 4 positive nodes or 1 to 3 nodes with grade 3 disease or tumour size > 5 cm or 1 to 3 positive nodes with Ki-67 of 20 % or more
Ki 67 cut off	No cut off but 75% patients had a Ki-67 of <15%	No cut off	20 % or more
Duration of treatment	1 year	2 years	2 years
